# Lack of association between particulate air pollution and blood glucose levels and diabetic status in peri-urban India

**DOI:** 10.1016/j.envint.2019.105033

**Published:** 2019-10

**Authors:** Ariadna Curto, Otavio Ranzani, Carles Milà, Margaux Sanchez, Julian D. Marshall, Bharati Kulkarni, Santhi Bhogadi, Sanjay Kinra, Gregory A. Wellenius, Cathryn Tonne

**Affiliations:** a ISGlobal, Universitat Pompeu Fabra, CIBER Epidemiología y Salud Pública, Barcelona, Spain; b Department of Civil and Environmental Engineering, University of Washington, WA, USA; c National Institute of Nutrition, Indian Council of Medical Research, Hyderabad, India; d Public Health Foundation of India, Indian Institute for Public Health, Hyderabad, India; e Department of Non-communicable Disease Epidemiology, London School of Hygiene and Tropical Medicine, London, UK; f Department of Epidemiology, Brown University School of Public Health, RI, USA

**Keywords:** PM_2.5_, particles with a diameter ≤ 2.5 μm, BC, black carbon, T2DM, Type 2 diabetes mellitus, APCAPS, Andhra Pradesh Children and Parents Study, GIS, Geographical Information System, NIN, National Institute of Nutrition, WHO, World Health Organization, LUR, land-use regression, BMI, body mass index, FFQ, food frequency questionnaire, MET, metabolic equivalent task, SES, socio-economic status, SLI, standard of living index, IQR, inter-quartile range, ETS, environmental tobacco smoke, CI, confidence interval, OR, odds ratio, SD, standard deviation, Blood glucose, Prediabetes, Diabetes, Air pollution, Particulate matter, Black carbon

## Abstract

**Background:**

Limited evidence exists on the effect of particulate air pollution on blood glucose levels. We evaluated the associations of residential and personal levels of fine particulate matter (PM_2.5_) and black carbon (BC) with blood glucose and diabetic status among residents of 28 peri-urban villages in South India.

**Methods:**

We used cross-sectional data from 5065 adults (≥18 years, 54% men) included in the Andhra Pradesh Children and Parents Study. Fasting plasma glucose was measured once in 2010–2012 and prevalent prediabetes and diabetes were defined following the American Diabetes Association criteria. We estimated annual ambient PM_2.5_ and BC levels at residence using land-use regression models and annual personal exposure to PM_2.5_ and BC using prediction models based on direct measurements from a subsample of 402 participants. We used linear and logistic nested mixed-effect models to assess the association between exposure metrics and health outcomes. For personal exposures, we stratified analyses by sex.

**Results:**

Mean (SD) residential PM_2.5_ and BC were 32.9 (2.6) μg/m^3^ and 2.5 (2.6) μg/m^3^, respectively; personal exposures to PM_2.5_ and BC were 54.5 (11.5) μg/m^3^ and 5.8 (2.5) μg/m^3^, respectively. Average (SD) fasting blood glucose was 5.3 (1.3) mmol/l, 16% of participants had prediabetes, and 5.5% had diabetes. Residential PM_2.5_ and BC were not associated with higher blood glucose levels. Personal PM_2.5_ (20 μg/m^3^ increase) and BC (1 μg/m^3^ increase) were negatively associated with blood glucose levels in women (PM_2.5_: −1.93, 95%CI: −3.12, −0.73; BC: −0.63, 95%CI: −0.90, −0.37). In men, associations were negative for personal PM_2.5_ (−1.99, 95%CI: −3.56, −0.39) and positive for personal BC (0.49, 95%CI: −0.44, 1.43). We observed no evidence of associations between any exposure and prevalence of prediabetes/diabetes.

**Conclusions:**

Our results do not provide evidence that residential exposures to PM_2.5_ or BC are associated with blood glucose or prevalence of prediabetes/diabetes in this population. Associations with personal exposure may have been affected by unmeasured confounding, highlighting a challenge in using personal exposure estimates in air pollution epidemiology. These associations should be further examined in longitudinal studies.

## Highlights

• We evaluated long-term air pollution exposure in adults from peri-urban India.
• Ambient air pollution was not associated with higher blood glucose levels.
• Personal air pollution exposure was associated with lower blood glucose levels.
• We observed no associations between PM_2.5_/BC and prevalence of prediabetes/diabetes.

## 1 Introduction

Over the last decade, air quality has improved in some high-income regions but worsened in many low- and middle-income regions such as South Asia (Brauer et al., 2016; Health Effects Institute, 2018). Similarly, Type 2 diabetes mellitus (T2DM) prevalence varies around the world. The majority (80%) of prevalent cases of T2DM are in less developed regions, particularly in Asia (Ramachandran et al., 2010). India and China are expected to have the largest number of people with T2DM in the world by 2030 (Wild et al., 2004).

Emerging evidence suggests that long-term air pollution may be associated with higher levels of fasting blood glucose (Thiering et al., 2013; Wolf et al., 2016; Chen et al., 2016; Thiering et al., 2016; Khafaie et al., 2018; Li et al., 2018; Toledo-Corral et al., 2018; B.Y. Yang et al., 2018; Dang et al., 2018). The observed associations have been generally stronger for gaseous pollutants than particulate pollutants (Wolf et al., 2016; B.Y. Yang et al., 2018; Dang et al., 2018). However, results from studies assessing fine particulate matter (PM_2.5_) are heterogeneous; with some studies (Wolf et al., 2016; B.Y. Yang et al., 2018) and a pooled analysis (Dang et al., 2018) suggesting weak or null associations.

Long-term exposure to particulate air pollution has also been linked to higher risk of development of T2DM (Rao et al., 2015; Kolb and Martin, 2017; Dendup et al., 2018; Alderete et al., 2018; Dang et al., 2018). Recent epidemiological evidence suggests that long-term exposure to PM_2.5_ is associated with T2DM incidence (Hansen et al., 2016; Requia et al., 2017; Qiu et al., 2018; Bowe et al., 2018) and prevalence (Qiu et al., 2018; Liu et al., 2016; Honda et al., 2017; Mazidi and Speakman, 2017; B.Y. Yang et al., 2018; Y. Yang et al., 2018). Although positive associations are supported by various pooled analyses (Balti et al., 2014; Janghorbani et al., 2014; Wang et al., 2014; Eze et al., 2015a; He et al., 2017), not all existing evidence has reported positive associations (Rao et al., 2015; Puett et al., 2010; Dzhambov and Dimitrova, 2016; Strak et al., 2017; O'Donovan et al., 2017; Renzi et al., 2018).

The current literature on the association between air pollution and T2DM and its traits is limited to North America and Europe (Balti et al., 2014; Janghorbani et al., 2014; Eze et al., 2015a; Dendup et al., 2018; Alderete et al., 2018) and urban areas in China (Brook et al., 2016; Jiang et al., 2016; Qiu et al., 2018; B.Y. Yang et al., 2018; Y. Yang et al., 2018). There is a need to expand research to highly exposed populations living in rural or peri-urban areas where the sources and chemical makeup of particles may differ relative to urban areas.

In addition, there is a need to study more specific markers of combustion-related particles such as black carbon (BC). Exposure to BC has been linked to cardiovascular and cardiorespiratory health (Hoek et al., 2013; Luben et al., 2017; Magalhaes et al., 2018), but to a lesser extent to metabolic health (Wolf et al., 2016; Strak et al., 2017; Renzi et al., 2018; Rajkumar et al., 2018). Most of these studies have been conducted in urban areas of high-income countries, where BC is a good indicator of road traffic emissions, particularly from diesel-powered vehicles (Querol et al., 2013). In contrast, in rural areas where use of unclean fuels for household energy (e.g., firewood, charcoal) is prevalent, BC is a good indicator of emissions from biomass burning (Reid et al., 2005).

Examining the association between long-term PM_2.5_ and BC and blood glucose levels in South Asians is particularly relevant. South Asians are exposed to high levels of indoor and outdoor air pollution and have a high-risk metabolic profile. They are particularly susceptible to insulin resistance, diabetes, and cardiovascular diseases (Unnikrishnan et al., 2014) and develop diabetes at earlier ages than do white Europeans (Sattar and Gill, 2015). We investigated whether residential or personal levels of PM_2.5_ and BC were associated with blood glucose or diabetic status among South Asian adults living in peri-urban India.

## 2 Materials and methods

### 2.1 Study population and ethics

**Fig. 1:**
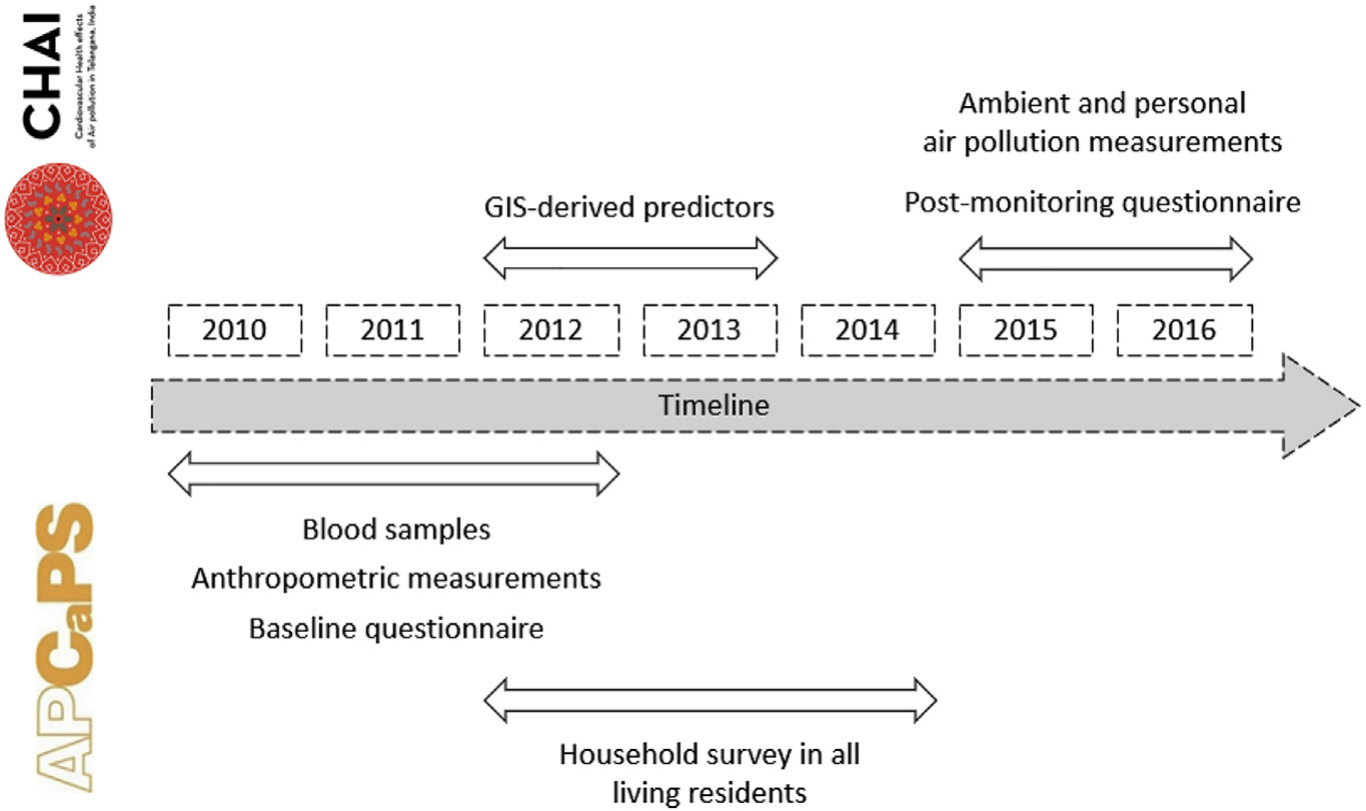
Timeline (in years) of data collection as part of the CHAI and APCAPS studies.

This study uses cross-sectional data from the third follow-up of the Andhra Pradesh Children and Parents Study (APCAPS). APCAPS is an intergenerational study in South India composed of adults born in 1987–1990 together with their parents and siblings (Kinra et al., 2014). The third follow-up of APCAPS was conducted between 2010 and 2012 (Fig. 1) and included questionnaire and health data for 6227 adults aged ≥18 years (excluding pregnant women). APCAPS participants lived in 28 villages in a peri-urban area (22 km × 35 km) southeast of Hyderabad city, Telangana (Fig. S1).

APCAPS was approved by the London School of Hygiene & Tropical Medicine (London, UK) and the National Institute of Nutrition (NIN) (Hyderabad, India). Signed consent forms were obtained from all participants.

### 2.2 Outcome assessment and definition

We collected one blood sample per participant in clinics established in the study villages as part of APCAPS. Participants attended morning clinics and were instructed to fast overnight and asked to sit for 10 min before venous blood withdrawal. Fasting plasma glucose was measured on the same day of blood collection using the glucose oxidase/peroxidase-4-aminophenazone-phenol (GOD-PAP) enzymatic method (Trinder, 1969). Ambient temperature and relative humidity were measured at Hyderabad International Airport weather station (Fig. S1) (data publicly available).

Definitions for prediabetes and for diabetes are as follows. We defined prediabetes using the criteria of the American Diabetes Association (American Diabetes Association, 2014) (i.e., fasting glucose ≥5.6 mmol/l and < 7 mmol/l). We defined diabetes (yes/no) when fasting glucose was ≥7 mmol/l (American Diabetes Association, 2014) and/or participant either having self-reported diabetes or self-reported physician-diagnosed diabetes. Since we could not distinguish between Type 1 and Type 2 diabetes, we present the overall prevalence of diabetes.

### 2.3 Air pollution assessment

We used estimates of annual average concentrations of PM_2.5_ and BC. Ambient monitoring was conducted during 2015–2016 within the framework of the CHAI project (Fig. 1), as previously described (Tonne et al., 2017). We collected 24-h gravimetric samples in 23 fixed-monitoring sites in the study area for a total of 21 days over two seasons (i.e., post-monsoon and summer) (Sanchez et al., 2018). Annual average concentrations at participants' residential address were estimated with land-use regression (LUR) models using geographic variables available for 2012–2013 (Fig. 1). The LUR models explained 58% of the variability in annual PM_2.5_ concentrations and 79% variability in the BC concentrations. Further details of ambient monitoring, distribution of measured concentrations at monitoring sites, and LUR model development can be found elsewhere (Sanchez et al., 2018). CHAI was approved by the Ethics Committees of Parc de Salut MAR (Barcelona, Spain), the Indian Institute of Public Health (Hyderabad, India), and the NIN.

In secondary analysis, we used estimates of annual personal exposure to PM_2.5_ and BC as additional measures of exposure. We measured personal exposure twice during 2015–2016 (Fig. 1) in a subset of 402 participants (random sample stratified by sex and village) in two different seasons. Participants wore a gravimetric sampler for at least 24 h, obtaining 610 participant-days of PM_2.5_ measurements and 569 participant-days of BC measurements. We used 67 potential time-invariant predictors of exposure (including individual and household characteristics from baseline questionnaire and general time-activity patterns from post-monitoring questionnaire) (Fig. 1) to predict personal exposure to PM_2.5_ and BC in all participants. Spearman correlations between measured and predicted personal levels and main predictors of personal exposure to PM_2.5_ and BC are presented in Table S1. Briefly, the main predictors of personal PM_2.5_ exposure in men were active smoking, occupation, and time spent cycling, and in women were occupation of the head of the household, time near biomass stove and primary stove type. For BC, the main predictors in men were occupation, ambient levels, time spent working, and sedentary time, and in women were again time near biomass stove and primary stove type as well as time spent in a motorized vehicle. More details on the prediction model can be found elsewhere (Sanchez et al., 2019).

### 2.4 Covariates

Data on relevant covariates were collected through anthropometric measurements in the clinic and an interviewer-administered baseline questionnaire (available at: http://apcaps.lshtm.ac.uk/questionnaires/) (Fig. 1). We calculated body mass index (BMI) as measured weight (in kg) divided by the square of measured height (in m). We calculated waist-to-hip-ratio by dividing waist circumference (in cm) by hip circumference (cm). Dietary intake over the past year was evaluated through a semi-quantitative food frequency questionnaire (FFQ). We obtained average daily consumption of sugar and sweets, alcohol, fruits, and carbohydrates from the FFQ (Bowen et al., 2012). We also collected the type and duration of all physical activities performed the preceding week (including sleep and sedentary time). Each activity was characterized by its Metabolic Equivalent Task (MET) (Matsuzaki et al., 2015). Physical activity level was categorized into extremely inactive or sedentary (<1.7 MET-week), moderately active (≥1.7 to <2 MET-week), and vigorously active (≥2 MET-week). Socio-economic status (SES) was evaluated through education attainment (without any type of formal education / with either primary, secondary or tertiary education) and standard of living index (SLI) of the household, categorized into tertiles. SLI is a composite index based on assets and characteristics of the household, including vehicle ownership and use of clean cooking fuel (Kinra et al., 2014).

### 2.5 Data analysis

Participants with missing data on sex (n = 4), household ID (n = 89), glucose levels (n = 61), and estimates of residential exposure levels (n = 546) were excluded from the main analysis. Among those participants without self-reported or clinical diagnosis of diabetes, we excluded those with no information on fasting status at the time of blood draw (n = 109) and those who did not fast for at least 8 h before blood collection (n = 353), leaving 5065 participants for main analyses (81%). We used multiple imputation (n = 20) to replace missing values in covariates using the method of chained equations (Buuren and Groothuis-Oudshoorn, 2011) and pooled regression results using Rubin's rules (Rubin, 1987).

Given the multilevel nature of our data (2245 households nested within 28 villages), all regression models were built using nested mixed-effects models. The main results are expressed as within-village variation of PM_2.5_ or BC: we used the difference between the household exposure and the village mean exposure (within effect) as the main exposure while controlling for the mean exposure of the village (between effect) (see the Supplementary material for model details). This method is conceptually equivalent to considering village as a fixed effect (see Section 3.3), but has the advantage of adjusting for the between-group unobserved effects using fewer degrees of freedom (Bell and Jones, 2015).

We applied linear mixed regression models to assess the association between residential exposure to PM_2.5_ and BC and concentrations of fasting blood glucose. From this analysis, we excluded participants taking any type of anti-diabetic medication (insulin and/or oral drugs) (n = 86). Fasting blood glucose was log-transformed (natural log transformation) to improve normality of residuals. Results are expressed as a percent change in the geometric mean of the outcome ([eβc - 1] × 100; where β is the regression coefficient and c is the exposure increase) (Barrera-Gómez and Basagaña, 2015) for a 1 μg/m^3^ increase in residential PM_2.5_ and for a inter-quartile range (IQR) increase in residential BC (0.1 μg/m^3^).

We combined participants with prediabetes and diabetes to assess the association of residential PM_2.5_ and BC with prevalent prediabetes/diabetes using unconditional logistic mixed regression models. Prediabetes and diabetes were combined due to low numbers of participants with diabetes in some villages (Fig. S2). Results are expressed as adjusted odds ratios for a 1 μg/m^3^ increase in residential PM_2.5_ and for a IQR increase in residential BC (0.1 μg/m^3^).

We selected age and sex as a priori confounders given their strong association with all exposure metrics and outcomes. Selection of other covariates was based on previous evidence. We used three models with progressive confounder adjustment: i) models adjusted for age, sex, and village mean exposure (model 1); and ii) models further adjusted for intake of sugar and sweets (in g/day), physical activity level (extremely inactive or sedentary/moderately active/vigorously active), education attainment (with/without), alcohol consumption (in g/day), active smoking (current smoker/former or non-smoker), environmental tobacco smoke (ETS) in the household (yes/no), SLI (in tertiles), and primary cooking fuel (biomass/clean) (model 2, the main model). Since BMI, waist-to-hip-ratio and hypertension can be considered either confounders or potential causal intermediates between air pollution and diabetes, in an additional model we further adjusted model 2 for BMI (in kg/m^2^), waist-to-hip-ratio (in cm; Spearman correlation with BMI was 0.5), and physician-diagnosed hypertension (yes/no) (model 3). To explore sensitivity of our main analyses, we checked: the robustness of fasting status by excluding participants who did not fast for at least 12 h (n = 743) rather than 8 h, and the potential influence of between-village variability in confounding variables by dropping one village at a time from main model 2.

In secondary analyses, we used estimates of personal exposure to PM_2.5_ and BC as exposures. We included 5155 participants for personal analyses (83%). We added 90 participants with complete personal exposure data (but missing data on residential exposure). The distribution of personal exposure estimates was similar across villages if compared to ambient exposure estimates (Figs. S3 and S4) so confounding by unaccounted factors at the village level was unlikely. We therefore fitted mixed-effects models using the untransformed exposure without adjustment for the village-mean exposure.

Since prediction models of personal exposure were developed separately for men and women (Sanchez et al., 2019), we stratified secondary analyses by sex. We were interested in capturing the total personal exposure to particles regardless the source so we did not adjust for covariates representing sources of particulate air pollution (i.e. smoking, occupation, and type of primary cooking fuel). We fitted: i) models adjusted for age (model P1); ii) models further adjusted for sugar and sweets intake, physical activity level, education attainment and alcohol consumption (model P2); and iii) models further adjusted for BMI, waist-to-hip-ratio, and physician-diagnosed hypertension (model P3). For personal models, we expressed results as the percent change of 8-h fasting blood glucose level and as odds ratio for prevalent prediabetes/diabetes, all expressed as IQR increase in personal PM_2.5_ (20 μg/m^3^) and 1 μg/m^3^ increase in personal BC. All analyses were conducted in R (version 3.5.0) using packages “mice” (Buuren and Groothuis-Oudshoorn, 2011) and “lme4” (Bates et al., 2015).

## 3 Results

### 3.1 Descriptive characteristics

The 5065 participants included in main analysis were 38 (SD: 13) years old, predominantly men (54%), and predominantly without any type of formal education (53%) (Table 1). Most participants (58%) used biomass in their households as primary cooking fuel. Compared to men, women had less formal education, were physically more active, were non-smokers and consumed much less alcohol per day. We identified 16% of participants as meeting the criteria for prediabetes and 5.5% for diabetes (21% all together); prevalence was similar between men and women. Among participants with diabetes (n = 281), 39% were physician-diagnosed, 3.5% were taking insulin, 31% were taking anti-diabetic oral drugs, and 2.5% were taking both. Among participants with physician-diagnosed diabetes (n = 109), 25% did not know or remember the name of the medication they were taking. Fasting blood glucose levels did not correlate with ambient temperature (Pearson r: −0.05) or with relative humidity (Pearson r: −0.01) the same day of the blood collection. Averages of long-term residential PM_2.5_ and BC were 33 μg/m^3^ and 2 μg/m^3^, respectively. Averages of personal PM_2.5_ and BC were 54 μg/m^3^ and 6 μg/m^3^, respectively, with higher personal concentrations in women than men (Table 1).

**Table 1:** Participants' characteristics and levels of exposures and outcomes.

	All (n = 5065)	Men (n = 2719)	Women (n = 2346)
**Individual characteristics**			
Age (years), mean ± SD	37.5 ± 13.4	37.0 ± 14.9	38.0 ± 11.3
Formal education			
Without (either illiterate or literate)	2679 (52.9)	1029 (37.9)	1650 (70.3)
With any kind	2385 (47.1)	1689 (62.1)	696 (29.7)
Physical activity, n(%)			
Extremely inactive or sedentary	3146 (65.9)	1867 (72.4)	1279 (58.3)
Moderately active	1351 (28.3)	597 (23.1)	754 (34.4)
Vigorously active	274 (5.7)	115 (4.5)	159 (7.3)
BMI (kg/m^2^), mean ± SD	21.1 ± 3.8	20.9 ± 3.5	21.4 ± 4.0
Waist-to-hip ratio (cm), mean ± SD	0.9 ± 0.1	0.9 ± 0.1	0.8 ± 0.1
Smoking status, n(%)			
Never or former smoker	4250 (83.9)	1908 (70.2)	2342 (99.8)
Current smoker	814 (16.1)	810 (29.8)	4 (0.2)

**Household characteristics**			
Standard of living index tertiles, n(%)			
Low	1571 (33.5)	799 (31.6)	772 (35.7)
Medium	1569 (33.5)	843 (33.4)	726 (33.6)
High	1548 (33.0)	885 (35.0)	663 (30.7)
Environmental tobacco smoke, n(%)			
Yes	1599 (31.6)	722 (26.6)	877 (37.4)
No	3465 (68.4)	1986 (73.4)	1469 (62.6)
Primary cooking fuel, n(%)			
Clean (gas or electricity)	1961 (41.6)	1093 (42.9)	868 (39.9)
Biomass	2757 (58.4)	1452 (57.1)	1305 (60.1)
Distance to the primary road (km), mean ± SD	4.4 ± 2.8	4.5 ± 2.8	4.3 ± 2.8
Distance to the ring road (km), mean ± SD	9.5 ± 4.5	9.5 ± 4.5	9.5 ± 4.5

**Dietary profile**			
Alcohol intake (g/day), mean ± SD	83.9 ± 166.6	125.1 ± 205.7	36.1 ± 81.4
Sugar and sweets intake (g/day), mean ± SD	22.3 ± 19.5	23.0 ± 19.7	21.5 ± 19.2
Fat intake (g/day), mean ± SD	43.2 ± 23.9	48.0 ± 26.2	37.7 ± 19.6
Fruit intake (g/day), mean ± SD	132.0 ± 141.2	145.4 ± 154.9	116.5 ± 121.7
Carbohydrates intake (g/day), mean ± SD	387.3 ± 161.9	438.4 ± 177.3	328.2 ± 116.8
Vegetarian, n(%)	111 (2.2)	43 (1.6)	68 (2.9)

**Cardiometabolic profile**^a^			
Fasting glucose (mmol/l), mean ± SD	5.3 ± 1.3	5.3 ± 1.3	5.2 ± 1.3
Diabetes, n(%)	281 (5.5)	161 (5.9)	120 (5.1)
Prediabetes, n(%)	816 (16.1)	458 (16.8)	358 (15.3)
Self-reported diabetes, n(%)	138 (2.7)	82 (3.0)	56 (2.4)
Physician-diagnosed diabetes, n(%)	109 (2.2)	61 (2.3)	48 (2.1)
Taking any type of anti-diabetic medication, n(%)	87 (1.7)	44 (1.6)	43 (1.8)
Physician-diagnosed hypertension, n(%)	298 (6.1)	165 (6.3)	133 (5.9)

**Air pollution concentrations**			
Ambient PM_2.5_ (μg/m^3^), mean ± SD	32.9 ± 2.6	32.9 ± 2.6	32.9 ± 2.7
Personal PM_2.5_ (μg/m^3^), mean ± SD	54.5 ± 11.5	49.8 ± 8.9	60.1 ± 11.8
Ambient BC (μg/m^3^), mean ± SD	2.5 ± 0.2	2.5 ± 0.2	2.5 ± 0.2
Personal BC (μg/m^3^), mean ± SD	5.8 ± 2.5	4.4 ± 0.8	7.5 ± 2.7

SD: standard deviation; BMI: body mass index; PM_2.5_: particles <2.5 μm in diameter; BC: black carbon.

a For personal dataset, data are reported for 5155 participants with available personal data (2801 men and 2354 women). Diabetes prevalence among participants with personal data was 5.0% (n = 258) and prevalence of prediabetes was 15.8% (n = 813).

### 3.2 Main results

Within-village residential concentrations of PM_2.5_ and BC were not statistically significantly associated with 8-h fasting blood glucose levels in any model (Table 2). For reference, crude effects are shown in Table S2. In model 2, we estimated an increase of 0.54% of 8-h fasting blood glucose (95% Confidence Interval (95%CI): −0.77%, 1.86%) for a 1 μg/m^3^ increase in within-village PM_2.5_. For within-village BC, point estimates were smaller, although CIs were narrower. Associations between residential exposure to PM_2.5_ and BC and prevalence of prediabetes/diabetes were consistent with the null hypothesis of no association (Table 2).

**Table 2:** Associations between residential exposure to PM_2.5_ and black carbon (BC) with blood glucose levels and prevalence of prediabetes/diabetes.

	All participants		
	Model 1^a^	Model 2^b^	Model 3^c^
Blood glucose	% change (95%CI)	% change (95%CI)	% change (95%CI)

8-h fasting (n = 5065)			
PM_2.5_	0.39 (−0.91; 1.71)	0.54 (−0.77; 1.86)	0.48 (−0.78; 1.76)
BC	0.29 (−0.35; 0.92)	0.33 (−0.30; 0.97)	0.34 (−0.28; 0.95)
12-h fasting (n = 4322)			
PM_2.5_	0.56 (−0.79; 1.93)	0.72 (−0.64; 2.08)	0.65 (−0.66; 1.98)
BC	0.34 (−0.31; 1.01)	0.40 (−0.25; 1.07)	0.39 (−0.25;1.03)

Prevalence of prediabetes/diabetes^d^	OR (95%CI)	OR (95%CI)	OR (95%CI)

8-h fasting (n = 5065)			
PM_2.5_	0.98 (0.81; 1.19)	0.99 (0.82; 1.21)	0.99 (0.81; 1.20)
BC	1.05 (0.95; 1.15)	1.06 (0.96; 1.16)	1.06 (0.96; 1.17)
12-h fasting (n = 4322)			
PM_2.5_	0.94 (0.77; 1.16)	0.96 (0.78; 1.19)	0.96 (0.78; 1.19)
BC	1.00 (0.90; 1.11)	1.01 (0.91; 1.12)	1.02 (0.91; 1.13)

Results are expressed as percent change of 8-h and 12-h fasting blood glucose concentrations, adjusted odds ratio (OR) for prevalence of prediabetes/diabetes, and corresponding 95% confidence intervals (95%CI) per 1 μg/m^3^ increase in within-village fine particulate matter (PM_2.5_) and 0.1 μg/m^3^ increase in within-village BC.

a Model 1: outcome ~ PM_2.5_/BC residual + age + sex + mean PM_2.5_/BC village + (1|village/household).

b Model 2 (main model): model 1 + sugar and sweets intake + physical activity + education + alcohol intake + smoking + environmental tobacco smoke + standard of living index + cooking fuel.

c Model 3: model 2 + body mass index + waist-to-hip-ratio + physician-diagnosed hypertension.

d Diabetes was defined as fasting blood glucose ≥ 7 mmol/l and/or participant either having self-reported diabetes or physician-diagnosed diabetes. Prediabetes was defined as fasting blood glucose ≥ 5.6 mmol/l and <7 mmol/l.

### 3.3 Sensitivity of main results

Results were similar in sensitivity analyses restricted to participants having fasted for at least 12 h (instead of 8 h) (Table 2). Results were also similar in sensitivity analyses treating village as a categorical fixed effect (Table S3).

To gain further insights into whether the results of our main analyses (Model 2) were driven by a small number of villages we repeated the main analysis leaving out one village at a time (Figs. S5, S6). For PM_2.5_, results varied somewhat when excluding villages 14 and 33 from analysis, but the overall conclusions were unchanged. For BC, results were consistent with the model including all villages.

### 3.4 Secondary results

In the analysis between personal exposure and glucose levels (Table S4), we observed statistically significant associations between higher personal exposure to PM_2.5_ (20 μg/m^3^ increase) and BC (1 μg/m^3^ increase) and lower blood glucose in women (PM_2.5_: −1.93%, 95%CI: −3.12%, −0.73%; BC: −0.63%, 95%CI: −0.90%, −0.37%). In men, only positive associations were found for personal BC (0.49%, 95%CI: −0.44%, 1.43%).

In the analysis between personal exposure and prevalence of prediabetes/diabetes, associations were generally negative and included the null in both sexes (Table S4); except for the association of personal BC in women, which was negative and statistically significant (OR model 2: 0.93; 95%CI: 0.89; 0.98).

## 4 Discussion

We evaluated associations between residential and personal exposure to PM_2.5_ and BC with fasting blood glucose and prevalence of prediabetes/diabetes in a peri-urban population living in South India. We observed that residential PM_2.5_ and BC concentrations were not associated with concentrations of fasting blood glucose or with a higher prevalence of prediabetes/diabetes. Our results were consistent across a range of sensitivity analyses. In secondary analyses, we observed negative associations between personal exposure to PM_2.5_ and BC and blood glucose levels and null associations for prevalence of prediabetes/diabetes.

### 4.1 Comparison with previous studies

To the best of our knowledge, this is the first study to assess associations between long-term air pollution and diabetes in South Asia. O'Donovan and colleagues included 1839 participants from South Asian origin residing in the UK when assessing the association between ambient PM_2.5_ and prevalence of diabetes (O'Donovan et al., 2017). They found null associations comparable to those from our fully-adjusted model (i.e., adjusting by ethnicity, urban/rural location, physical activity and BMI, among others). To our knowledge, our study is also the first conducted in a non-urban area from a lower-middle-income country, where traffic is not explaining the spatial variation in ambient PM_2.5_ (Sanchez et al., 2018) or the personal exposure to PM_2.5_ (Milà et al., 2018). Our study area is mostly influenced by air pollution from household biomass burning, agricultural crop burning, and local industry (mostly rice mills and brick kilns) (Kumar et al., 2018). This mix of sources is what determines the composition and toxicity of particles and the associated health effects in this area, differentiating it from urban areas mostly dominated by traffic sources. This is the reason why in the following sections we compare our results with previous studies in China, which may be more comparable to our study in terms of sources (most are nationwide studies that include rural areas with high influence of smoke from solid fuel burning) and ambient levels (all reporting ambient PM_2.5_ levels ≥36 μg/m^3^).

### 4.2 Residential PM_2.5_

Our results do not provide evidence of an association between PM_2.5_ levels and blood glucose levels. This is consistent with a recent meta-analysis based on longitudinal studies that found a null relationship between ambient PM_2.5_ and levels of fasting blood glucose (0.02%, 95%CI: −0.05%, 0.08%) (Dang et al., 2018). There are some cross-sectional studies, however, that have observed positive associations (Chuang et al., 2011; Liu et al., 2016; B.Y. Yang et al., 2018). Chuang and colleagues studied a sample of 1023 middle-aged adults (≥54 years old) living in Taiwan and found a positive and strong association between higher county or city PM_2.5_ levels and higher levels of fasting blood glucose (36.6 mmol/l increase per 20 μg/m^3^ PM_2.5_ increase; 95%CI: 19.2, 53.9) (Chuang et al., 2011). Liu and colleagues conducted a nation-wide study among middle-aged (≥45 years old) Chinese adults, finding strong associations between PM_2.5_ and blood glucose either when considering satellite-based estimates (0.26 mmol/l per 41 μg/m^3^ PM_2.5_ increase; 95%CI: 0.20, 0.32) or modelled concentrations from the 2013 Global Burden of Disease study (0.22 mmol/l; 95%CI: 0.16, 0.28) (Liu et al., 2016). Yang and colleagues studied >15,000 adults (≥18 years old) living in three cities in northeast China. They found that each 26 μg/m^3^ increase in PM_2.5_ was associated with modestly higher levels of blood glucose (0.08 mmol/l; 95%CI: 0.04, 0.12) (B.Y. Yang et al., 2018).

Regarding the prevalence of prediabetes/diabetes, we found no evidence of associations with residential exposure to PM_2.5_ and BC. A handful of studies conducted in East Asia have supported an association between PM_2.5_ and prevalence of diabetes (Liu et al., 2016; Qiu et al., 2018; B.Y. Yang et al., 2018; Y. Yang et al., 2018). Yang and colleagues found an adjusted OR of 1.14 (95%CI: 1.03, 1.25) per each 26 μg/m^3^ increase in PM_2.5_ (B.Y. Yang et al., 2018). Liu and colleagues found a diabetes prevalence ratio of 1.14 (95%CI: 1.08, 1.20) for every 41.1 μg/m^3^ increase in PM_2.5_ (Liu et al., 2016). A study conducted in Hong Kong among older adults (≥65 years old), found an adjusted odds ratio of 1.05 (95%CI: 1.01, 1.10) per each 3.2 μg/m^3^ increase in ambient PM_2.5_ (Qiu et al., 2018). Another nation-wide study conducted among middle-aged (≥50 years old) Chinese adults, found an adjusted OR of prevalent diabetes of 1.27 (95%CI: 1.12, 1.43) per each 10 μg/m^3^ increase in ambient PM_2.5_ (Y. Yang et al., 2018).

There are several reasons that could explain the differences between our results and these Chinese studies. First, our sample size was smaller and our exposure range narrower, thus potentially reducing the precision of our estimates and limiting our ability to detect statistically significant associations. Second, the prevalence of diabetes reported in most of these studies was higher (>10%) than ours (5.5%), thus improving their statistical power. Third, these studies generally focused on older populations than our study population, who may be more vulnerable to the cardiometabolic health effects of air pollution.

In light of our null associations between air pollution and blood glucose levels and prevalence of prediabetes/diabetes, it could be the case that our high concentration levels are in the flat part of the concentration-response curve, as previously seen for PM_2.5_ and incident diabetes (Bowe et al., 2018). The curve presented by Bowe et al. showed that the risk of diabetes moderately increases at concentrations of PM_2.5_ above 10 μg/m^3^ and reach a plateau at 13 μg/m^3^, concentration much lower than the one found in our study for ambient PM_2.5_ (33 μg/m^3^). This concentration-response curve should be confirmed in future longitudinal studies with PM_2.5_ concentrations higher than 17 μg/m^3^, where large uncertainty remains.

### 4.3 Residential black carbon

We also found null associations for residential BC. We are not aware of prior studies exploring the role of long-term residential exposure to BC in regards to metabolic health. However, few European studies have explored long-term levels of PM_2.5_ absorbance, a comparable measure to BC (Wolf et al., 2016; Strak et al., 2017; Renzi et al., 2018). These studies found similar estimates as our study. Wolf and colleagues found that higher PM_2.5_ absorbance was weakly and modestly associated with higher fasting blood glucose in southern Germany (0.6%, 95%CI: 0.0%, 3.3%). Strak and colleagues studied a large sample of adults (≥19 years old) from the Dutch national health survey and found that higher PM_2.5_ absorbance was significantly but modestly associated with higher prevalence of diabetes (OR: 1.04, 95%CI: 1.02, 1.06). Renzi et al. studied more than one million adults (≥35 years old) in Italy and reported a negative and weak association between PM_2.5_ absorbance and prevalence of diabetes (Renzi et al., 2018). Renzi and colleagues partly attributed this lack of an association to the inability to control for emission sources other than traffic, such as domestic heating. In our study, the BC LUR model captured local sources of BC such as wood/gas supply places (Sanchez et al., 2018) and our models adjusted by primary cooking fuel. However, it may be the case that we were not able to capture the complexity of the mixture of sources in this peri-urban area.

### 4.4 Personal exposure to PM_2.5_ and black carbon

To our knowledge, this is the first study evaluating long-term personal exposure to PM_2.5_ and BC with blood glucose or diabetic status. Few prior studies have used short- or mid-term direct personal measurements in urban China (Brook et al., 2016; Jiang et al., 2016) and rural Honduras (Rajkumar et al., 2018). Brook et al. studied a sample of 65 adults with the metabolic syndrome in Beijing and found null associations between personal BC and blood glucose levels in lags from one to five days. Jiang et al. studied 371 adults living in Shanghai and only found significant associations between 10-h personal PM_2.5_ and blood glucose levels for those participants living between 50 and 100 m from a major road (1.32 mmol/l; 95%CI: 1.04, 1.61). Rajkumar et al. assessed 24-h personal PM_2.5_ and BC among 142 Honduran women, observing positive associations between personal PM_2.5_ and BC and prevalence of prediabetes/diabetes (prevalence ratios of 1.49; 1.11, 2.01, for every 84 μg/m^3^ increase in personal PM_2.5_ and of 1.26; 1.13, 1.40, for every 14 μg/m^3^ increase in personal BC).

### 4.5 Potential biological mechanisms

Insulin resistance is the hallmark biological pathway for the development of diabetes and has also been suggested as the strongest pathway by which air pollution can affect cardiometabolic health (Eze et al., 2015b). Some of the potential pathways by which PM_2.5_ could increase insulin resistance are endothelial dysfunction or vasoconstriction caused by oxidative stress, stimulation of the sympathetic nervous system, and/or the creation of a systemic pro-inflammatory state (e.g., by releasing the cytokines tumor necrosis factor-alpha (TNF-α) and interleukin-6 (IL-6)), among other pathways previously discussed elsewhere (Brook and Rajagopalan, 2009; Rajagopalan and Brook, 2012; Rao et al., 2015). Other pathways by which PM_2.5_ and other air pollutants could contribute to the development of diabetes include impaired renal function (Mehta et al., 2016), obesity and weight gain (Li et al., 2016), and alterations in mitochondria and brown adipose tissue (Rajagopalan and Brook, 2012).

### 4.6 Potential biases and statistical power

In addition to a true lack of a causal effect of air pollution on glucose homeostasis, there are a number of additional potential explanations as to why we did not find positive associations between air pollution and markers of T2DM. First, the ambient and personal air pollution data used in LUR and personal prediction models to assess exposure were collected between 3 and 6 years after (2015–2016) the assessment of health outcomes and the baseline questionnaire (2010−2012) (Fig. 1). Our results are valid under the assumption that estimated air pollution levels were representative of exposure on the order of a few years. This assumption is plausible for residential levels where the spatial pattern of air pollutants is unlikely to change over periods of 10–15 years (Gulliver et al., 2011). As discussed in Sanchez et al. (Sanchez et al., 2018), we believe that the temporal discrepancy between the GIS-derived predictors and the air pollution monitoring measurements used for LUR development possibly had little impact on the performance of our LUR models. In contrast with ambient levels, personal levels could have higher variability over time. However, most of the predictors of personal exposure were collected through the baseline questionnaire at the time of outcome measurement (2010–2012).

Second, our analyses may have suffered from limited statistical power due to the relatively small variability in within-village exposure and low prevalence of diabetes across villages. As a result, we obtained fairly wide confidence intervals (particularly for PM_2.5_ estimates), which may have precluded us from finding small (perhaps clinically important) positive associations.

Third, our study population was relatively young (mean 38 years) and lean (mean BMI 21 kg/m^2^), and this perhaps make it at low risk of developing diabetes. On the other hand, it has been shown that South Asians, when compared to other ethnicities, progress to the high-risk prediabetes phase earlier and are diagnosed with diabetes at younger ages (5–10 years earlier) and at lower BMI (South Asians are considered at an increased risk of developing diabetes at BMI of 23 kg/m^2^) (Sattar and Gill, 2015).

Fourth, negative associations observed for personal exposures may indicate that we did not have data on all relevant confounders or likely measurement error in personal behaviors influencing exposure (e.g., physical activity), potentially increasing the bias in the health effects from unmeasured confounding and/or reverse causation (Weisskopf and Webster, 2017). Personal exposure was more highly correlated with individual characteristics predictive of health (e.g., age, physical activity) than residential levels (Table S5). The trade-off between improved exposure measurement error and higher likelihood of confounding with personal compared to residential-based measures of exposure has been previously described (Weisskopf and Webster, 2017). We hypothesize that our evaluation of physical activity could have been one of the drivers of confounding. The questionnaire's ability to quantify the amount of light and sedentary activities is limited due to recall (Matsuzaki et al., 2015). Light activities are beneficial for blood glucose levels (Healy et al., 2007) and extended sedentary time is a metabolic risk factor that should be included as an independent confounder in future studies (Colberg et al., 2016).

Fifth, we cannot rule out some outcome measurement error. Fasting glucose level assessment was based on a single blood collection. More than one blood sample would have likely reduced potential outcome misclassification. Since misclassification was likely non-differential, it possibly produced bias towards the null. Although fasting blood glucose is the most extensively used test for its low cost and availability, it can underestimate the prevalence of prediabetes and diabetes if compared to the oral glucose tolerance test or measures of glycated hemoglobin (HbA1c) (Gerstein, 2001; Jeon et al., 2013), which were not available in APCAPS.

Finally, similar to most previous studies, we were unable to distinguish between Type 1 and Type 2 diabetes. However, most of the prevalent diabetic cases are likely Type 2 (90–95% of all diabetes) (American Diabetes Association, 2014); in our study, only 4% of physician-diagnosed participants were diagnosed before 25 years of age.

### 4.7 Strengths and limitations

The main strength of our contribution lies in the study population and the exhaustive air pollution exposure assessment, including the characterization of personal exposure, which is very uncommon in air pollution epidemiological literature. Our study also overcomes some limitations of previous studies such as the use of LUR models to allow the study of small spatial contrasts in exposure, the inclusion of BC as a better indicator of combustion-related sources than PM_2.5_ and the consideration of a wide range of covariates, including dietary intake, physical activity, and indoor air pollution indicators, not all considered in previous studies (Chuang et al., 2011; Liu et al., 2016; Qiu et al., 2018; B.Y. Yang et al., 2018; Y. Yang et al., 2018). An additional strength of our study is that the study population is representative of the general population in the study villages. We observed comparable demographic characteristics (e.g., age, sex, education) between our study participants and all surveyed adults from the general population (≈100%) (Curto et al., 2019).

The main limitations of this study are discussed in Subsection 4.6, namely exposure and outcome misclassification, limited statistical power and residual confounding. Our study is cross-sectional and therefore may be subject to reverse causality; e.g., diseased participants could have changed lifestyle as a result of their disease or moved to villages nearer Hyderabad city, where there is more accessibility to health care services.

## 5 Conclusions

Our results do not support the hypothesis that increased levels of ambient or personal PM_2.5_ and BC are associated with higher levels of fasting glucose or higher prevalence of prediabetes/diabetes in this peri-urban area of India. The lack of an association for residential particle levels is consistent with some emerging evidence, including one meta-analysis. To corroborate these results, associations between air pollution and prediabetes/diabetes and its traits should be further examined in populations with similar characteristics while putting emphasis on longitudinal designs and objective assessments of participants' behaviors.

## Funding

This work was supported by grants 084674/Z from the Wellcome Trust, 336167 from the European Research Council, and RYC-2015-17402 to investigator CT from the Spanish Ministry of Economy and Competitiveness.

## Declaration of Competing Interest

None declared.

## Data Availability

Data is available through a formal collaborator request to APCAPS (see: http://apcaps.lshtm.ac.uk/apply-to-collaborate/). The computing code required to replicate the results reported is available by contacting the corresponding author.

## Appendix A Supplementary data

Supplementary material Image 1
