# Supplementary material for: Lack of association between particulate air pollution and blood glucose levels and diabetic status in peri-urban India
Source: Environ Int. 2019 Oct;131:105033. doi: 10.1016/j.envint.2019.105033 (PMC6718580; doi:10.1016/j.envint.2019.105033)
Supplement: Supplementary file 1 — Supplementary material [file mmc1.docx]

**SUPPLEMENTARY MATERIAL**

**Lack of association between particulate air pollution and blood glucose levels and diabetic status in peri-urban India**

Ariadna Curto, Otavio Ranzani, Carles Milà, Margaux Sanchez, Julian D Marshall, [Bharati Kulkarni, Santhi Bhogadi](http://scholar.google.com/citations?user=2xYnjEsAAAAJ&hl=en), Sanjay Kinra, Gregory A Wellenius, Cathryn Tonne

Number of pages: 13

Number of Tables: 5

Number of Figures: 6

Content:

- **Figure S1**: map of the study area, page 2
- **Extended version of Methods/Data analysis,** page 3
- **Table S1**: Components of personal exposure to PM_2.5_ and black carbon according to prediction models, page 4
- **Figure S2**: Number of participants per village according to their diabetic status, page 5
- **Figure S3**: Box plots of estimated PM_2.5_ and BC at personal level according to village, page 6
- **Figure S4**: Box plots of estimated PM_2.5_ and BC at residence according to village, page 7
- **Figure S5**: Associations of ambient PM_2.5_ and BC on blood glucose in leave-one-village-out model (sensitivity), page 8
- **Figure S6**: Associations of ambient PM_2.5_ and BC on prevalence of prediabetes/diabetes in leave-one-village-out model (sensitivity), page 9
- **Table S2**: Crude associations of ambient PM_2.5_ and BC on blood glucose and prevalence of prediabetes/diabetes, page 10
- **Table S3**: Main analyses with village as a fixed effect, page 11
- **Table S4**: Secondary analyses with estimates of personal PM_2.5_/BC, page 12
- **Table S5**: Ambient vs. personal estimates according to covariates, page 13

**Figure S1** Map of the study area near Hyderabad city, South India.


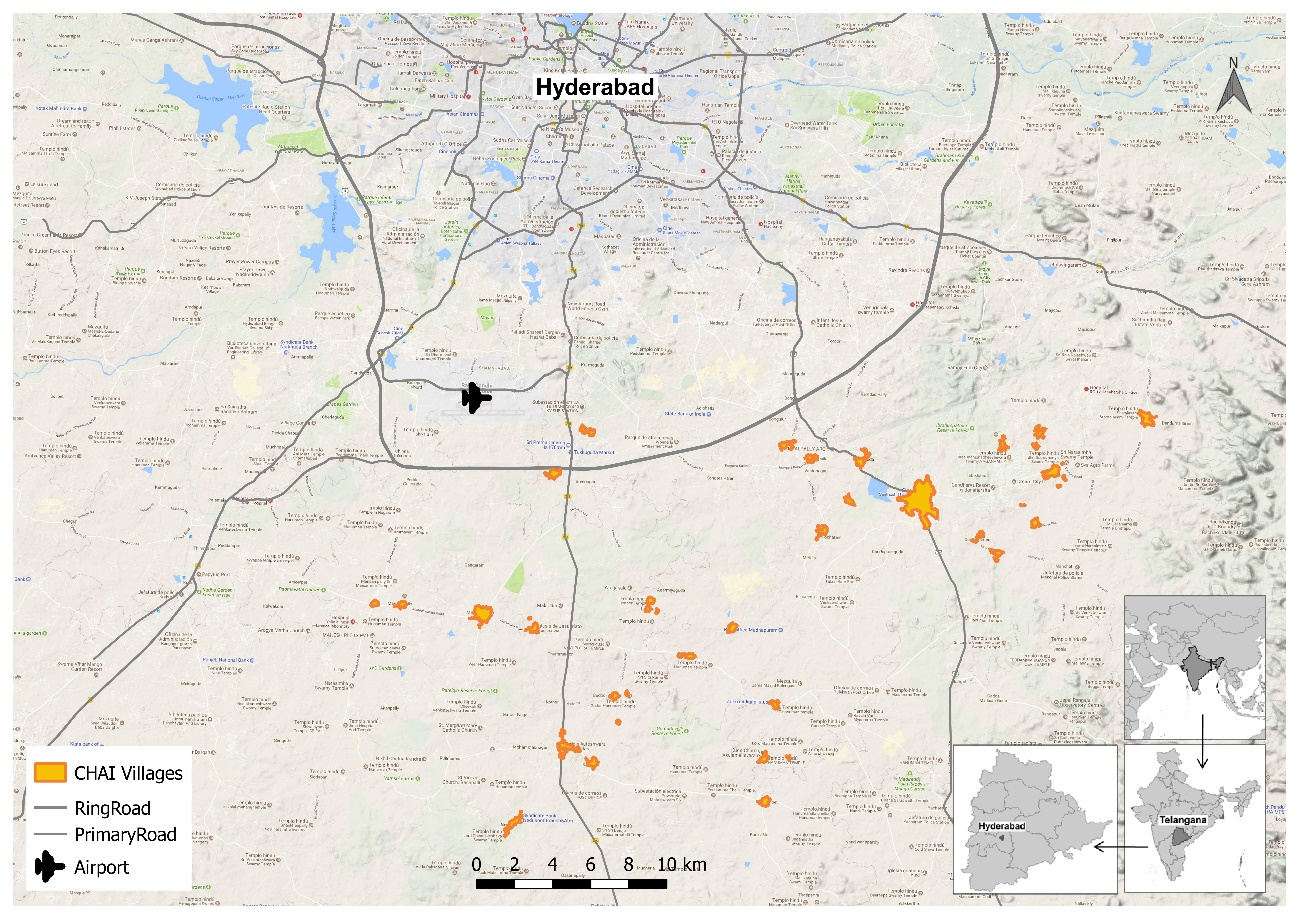


**MATERIALS AND METHODS (expanded version)**

**2.5 Data analysis**

To estimate within-village associations between ambient PM_2.5_ or BC and health, we applied nested mixed-effects models in which both the within and between village exposure-outcome relationships were modelled explicitly, an approach referred to as within-between model specification (Mundlak, 1978; Bell & Jones, 2015). We used the following regression equation (all components expressed in scalar form):

$$y_{vhi}= \beta_{0}+\beta_{w}\left( x_{vhi}-\bar{x}_{v} \right)+ \beta_{B}\bar{x}_{v}+(u_{v}+u_{vh}+e_{vhi}) + covariates$$

where $y_{vhi}$ represents the outcome in village $v,$ household $h$ and individual $i$; $\beta_{0}$ represents a constant; $\beta_{w}$ represents the within-village effect estimated as the effect of the difference between the individual exposure $\left( x_{vhi} \right)$ and the village mean $\left( \bar{x}_{v} \right)$ on the outcome; $\beta_{B}$ represents the village mean exposure (between effect); $u$ represent the random intercepts for the nested household ($u_{vh}$) within village ($u_{v}$); and $e_{vhi}$ the error term.

**References**:

Mundlak, Y. On the Pooling of Time Series and Cross Section Data. *Econometrica* **46**, 69 (1978). doi:10.2307/1913646

Bell, A. & Jones, K. Explaining Fixed Effects: Random Effects Modeling of Time-Series Cross-Sectional and Panel Data. *Polit. Sci. Res. Methods* **3**, 133–153 (2015). doi:10.1017/psrm.2014.7

**Table S1** Prediction models of personal exposure to PM_2.5_ and black carbon using time-invariant predictors.

|  |  | **Regression equation** | **R_spearman_** |
| --- | --- | --- | --- |
| PM_2.5_ | Men | α + α_participant_ – 21% if not smoking – 24% if passive smoking + 30% if construction or industry job – 12% per 1 hour spent cycling + 16% if unemployed + 4% if skilled manual occupation + 30% if unskilled manual occupation + ε_session_ | 0.31 |
|  | Women | α + α_participant_ + 13% per 1 hour spent cooking with biomass + 22% if biomass primary stove + 2% if unemployed household head + 23% if unskilled manual household head + ε_session_ | 0.42 |
| Black carbon | Men | α + α_participant_ + 38% office or shop job + 27% if biomass primary stove + 4% per 1 µg/m^3^ of residential ambient PM_2.5_ + ε_session_ | 0.30 |
|  | Women | α + α_participant_ + 62% if biomass primary stove + 20% per 1 hour spent cooking with biomass – 26% if motorcycle household ownership + ε_session_ | 0.50 |

*Abbreviations: α and α_participant_ are general intercept and participant-specific random intercept, respectively. The models include fixed and time-invariant predictors obtained from a baseline questionnaire. R_spearman_ represents the Spearman correlation coefficient between averaged measured and averaged predicted values per participant, among those with two measurements. Please, refer to Sanchez et al., 2019 for more details:*

Sanchez, M., Milà, C., Sreekanth, V., Balakrishnan, K., Sambandam, S., Nieuwenhuijsen, M., Kinra, S., Marshall, J.D., and Tonne, C. Personal exposure to particulate matter in peri-urban India: predictors and association with ambient concentration at residence. Journal of Exposure Science and Environmental Epidemiology (2019). doi: 10.1038/s41370-019-0150-5

**Figure S2** Number of participants per village according to their diabetic status.


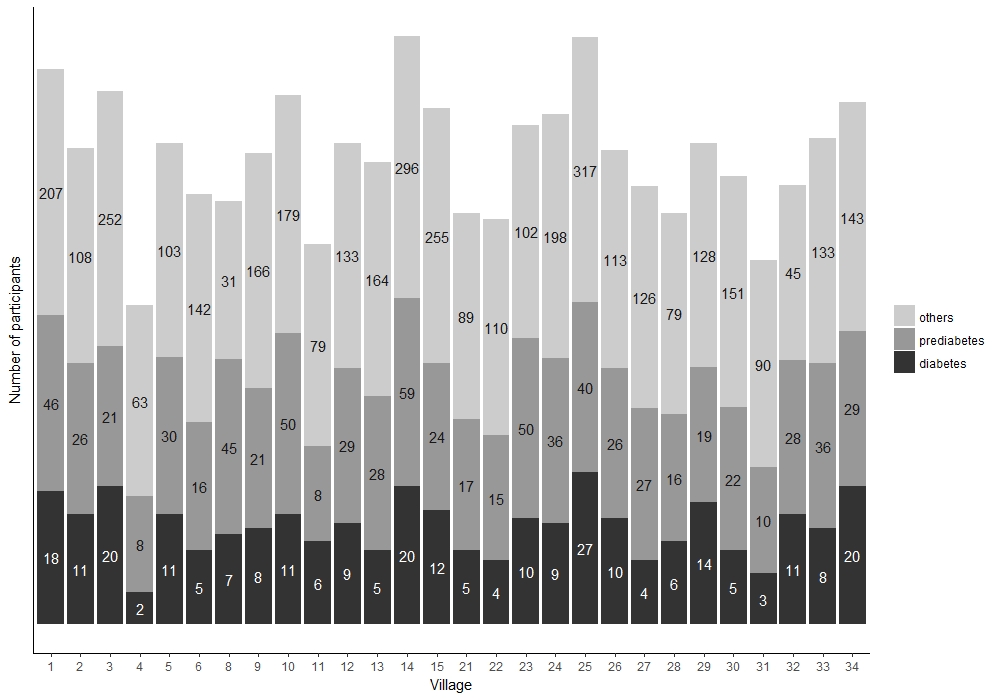


*Diabetes was defined as fasting blood glucose ≥7 mmol/l and/or participant either having self-reported diabetes or physician-diagnosed diabetes. Prediabetes was defined as fasting blood glucose ≥5.6 mmol/l and <7 mmol/l.*

**Figure S3** Box plots of estimated fine particulate matter (PM_2.5_) (panel A) and black carbon (BC) (panel B) at personal level according to village.


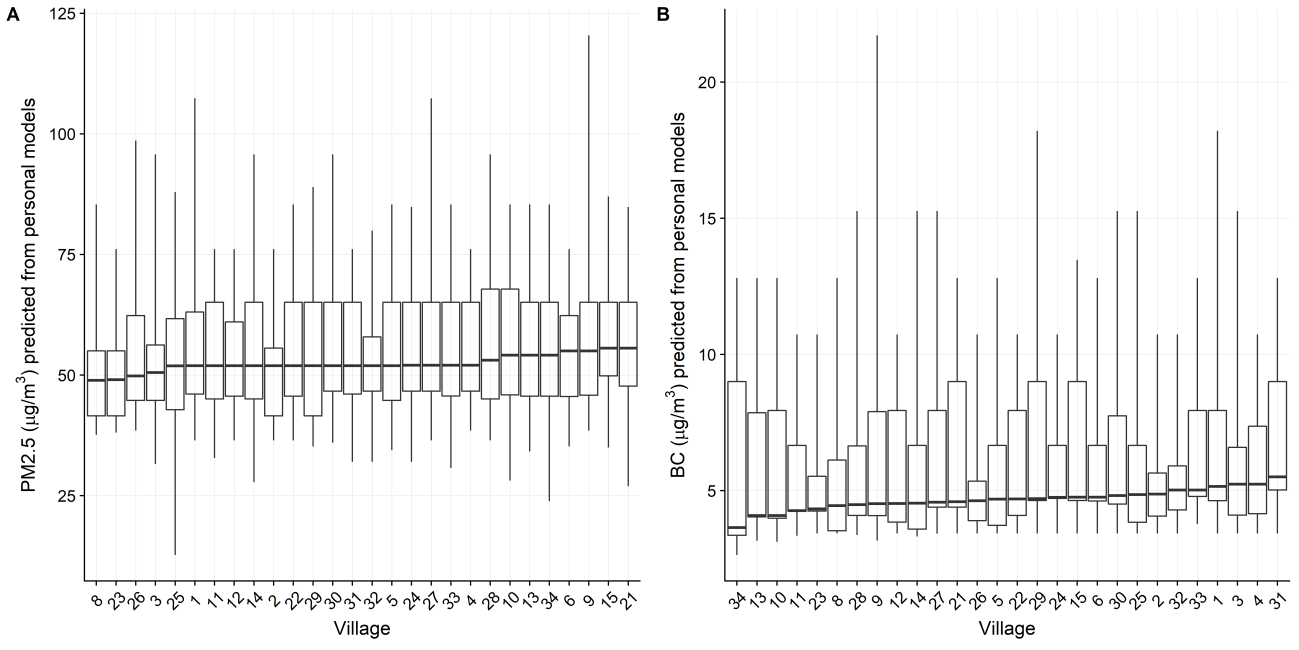


**Figure S4** Box plots of estimated fine particulate matter (PM_2.5_) (panel A) and black carbon (BC) (panel B) at residence according to village.

**
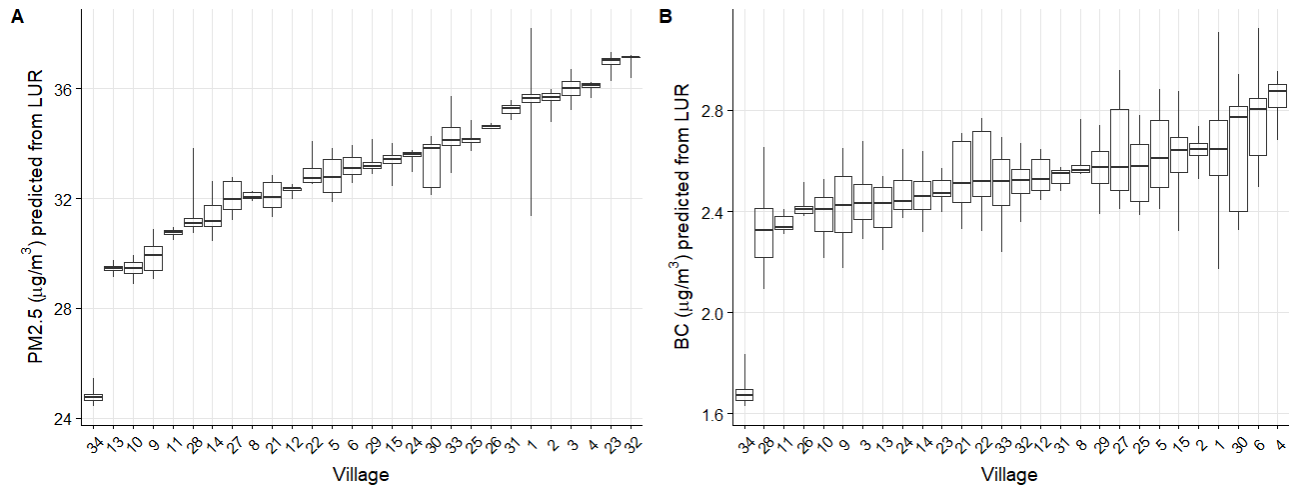
**

*LUR: Land-Use Regression model.*

**Figure S5** Percent change of fasting blood glucose concentration and 95% confidence intervals (95%CI) per 1 µg/m^3^ increase in within-village fine particulate matter (PM_2.5_) and 0.1 µg/m^3^ increase in within-village black carbon (BC) in leave-one-village-out models.


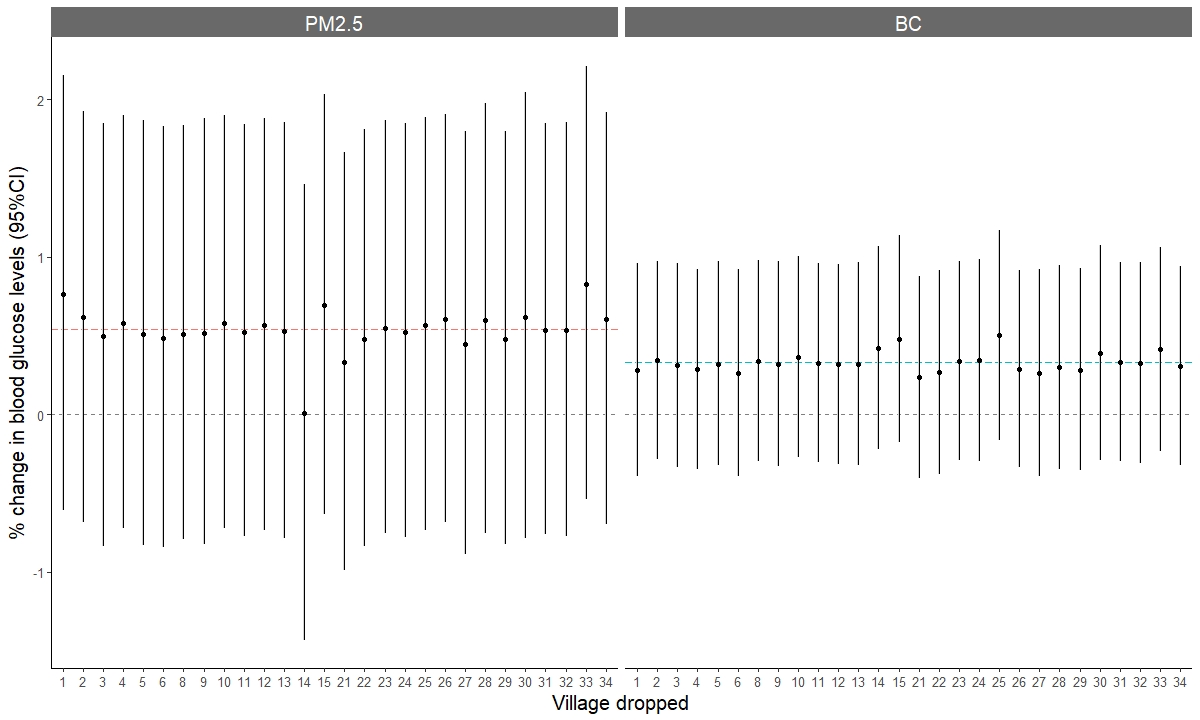


*Error bars represent 95% Confidence Interval. Dashed black line corresponds to the zero level. Red dashed line corresponds to the percent change obtained from the model 2 considering all villages (showed for reference) in PM_2.5_ model, whereas blue dashed line corresponds to BC model.*

**Figure S6** Adjusted odds ratio (OR) of prevalence of prediabetes/diabetes and 95% confidence intervals (95%CI) per 1 µg/m^3^ increase in within-village fine particulate matter (PM_2.5_) and 0.1 µg/m^3^ increase in within-village black carbon (BC) in leave-one-village-out models.


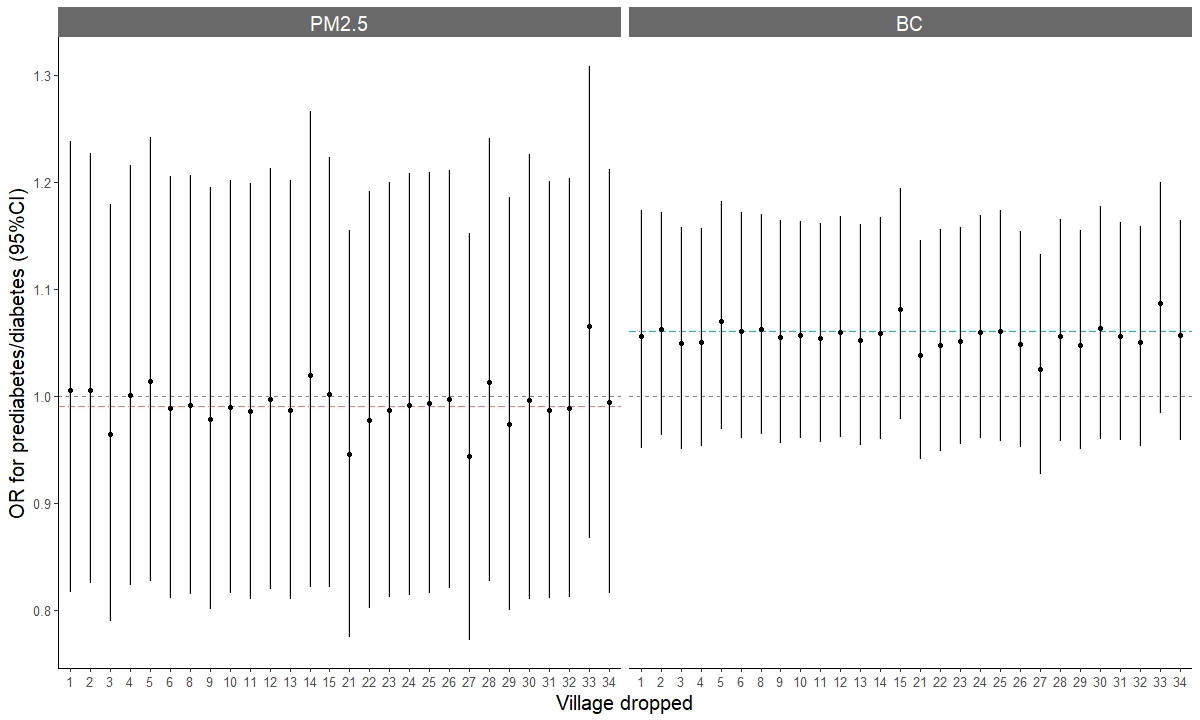


*Error bars represent 95% Confidence Interval. Dashed black line corresponds to the null level. Red dashed line corresponds to the percent change obtained from the model 2 considering all villages (showed for reference) in PM_2.5_ model, whereas blue dashed line corresponds to BC model.*

**Table S2** Associations between annual residential exposure to PM_2.5_ and black carbon (BC) with blood glucose levels and prevalence of prediabetes/diabetes showing crude models.

|  | ALL 8-h FASTING PARTICIPANTS (n= 5,065) | | | |
| --- | --- | --- | --- | --- |
|  | Crude^a^ | model 1^b^ | model 2^c^ | model 3^d^ |
| **Blood glucose** | % change  (95%CI) | % change  (95%CI) | % change  (95%CI) | % change  (95%CI) |
| PM_2.5_ | 0.37 (-0.94; 1.70) | 0.39 (-0.91; 1.71) | 0.54 (-0.77; 1.86) | 0.48 (-0.78; 1.76) |
| BC | 0.19 (-0.44; 0.84) | 0.29 (-0.35; 0.92) | 0.33 (-0.30; 0.97) | 0.34 (-0.28; 0.95) |
| **Prevalence of**  **prediabetes/diabetes**^d^ | OR  (95%CI) | OR  (95%CI) | OR  (95%CI) | OR  (95%CI) |
| PM_2.5_ | 0.98 (0.81; 1.18) | 0.98 (0.81; 1.19) | 0.99 (0.82; 1.21) | 0.99 (0.81; 1.20) |
| BC | 1.03 (0.94; 1.13) | 1.05 (0.95; 1.15) | 1.06 (0.96; 1.16) | 1.06 (0.96; 1.17) |

*Diabetes was defined as 8-h fasting blood glucose ≥7 mmol/l and/or participant either having self-reported diabetes or physician-diagnosed diabetes. Prediabetes was defined as 8-h fasting blood glucose ≥5.6 mmol/l and <7 mmol/l.*

1. Crude model: outcome ~ PM_2.5_/BC residual + (1 | village / household)
2. model 1: outcome ~ PM_2.5_/BC residual + age + sex + mean PM_2.5_/BC village + (1 | village / household)
3. model 2 (main model): model 1 + sugar and sweets intake + physical activity + education + alcohol intake + smoking + environmental tobacco smoke + standard of living index + cooking fuel
4. model 3: model 2 + body mass index + waist-to-hip-ratio + physician-diagnosed hypertension

**Table S3** Associations between residential exposure to PM_2.5_ and black carbon (BC) with blood glucose levels and prevalence of prediabetes/diabetes using village as a fixed effect.

|  | ALL 8-h FASTING PARTICIPANTS (n= 5,065) | | |
| --- | --- | --- | --- |
|  | model 1^a^ | model 2^b^ | model 3^c^ |
| **Blood glucose** | % change  (95%CI) | % change  (95%CI) | % change  (95%CI) |
| PM_2.5_ | 0.40  (-0.90; 1.72) | 0.54  (-0.76; 1.86) | 0.48  (-0.78; 1.76) |
| BC | 0.29  (-0.34; 0.93) | 0.33  (-0.30; 0.97) | 0.34  (-0.28; 0.95) |
| **Prevalence of**  **prediabetes/diabetes**^d^ | OR  (95%CI) | OR  (95%CI) | OR  (95%CI) |
| PM_2.5_ | 0.98  (0.80; 1.19) | 0.99  (0-82; 1.21) | 0.99  (0.81; 1.20) |
| BC | 1.05  (0.95; 1.16) | 1.06  (0.96; 1.16) | 1.06  (0.96; 1.17) |

*Results are expresses as percent change of 8-h fasting blood glucose concentration and odds ratio for prevalence of prediabetes/diabetes, and 95% confidence intervals (95%CI) per every 1 µg/m^3^ increase in within-village ambient fine particulate matter (PM_2.5_) and 0.1 µg/m^3^ increase in within-village ambient BC. Diabetes was defined as 8-h fasting blood glucose ≥7 mmol/l and/or participant either having self-reported diabetes or physician-diagnosed diabetes. Prediabetes was defined as 8-h fasting blood glucose ≥5.6 mmol/l and <7 mmol/l.*

1. model 1: outcome ~ LUR-derived PM_2.5_/BC + age + sex + village + (1 | household)
2. model 2: model 1 + sugar and sweets intake + physical activity + education + alcohol intake + smoking + environmental tobacco smoke + standard of living index + cooking fuel
3. model 3: model 2 + body mass index + waist-to-hip-ratio + physician-diagnosed hypertension

**Table S4** Associations between annual personal exposure to PM_2.5_ and black carbon (BC) with blood glucose levels and prevalence of prediabetes/diabetes.

|  | **Men (n=2,801)** | | | | **Women (n=2,354)** | | | |
| --- | --- | --- | --- | --- | --- | --- | --- | --- |
|  | Crude^a^ | model P1^b^ | model P2^c^ | model P3^d^ | Crude^a^ | model P1^b^ | model P2^c^ | model P3^d^ |
| **8-h fasting blood glucose** | | | | | | | | |
| **PM_2.5_** | 0.99  (-0.45; 2.46) | -2.45  (-3.98; -0.89) | -1.99  (-3.56; -0.39) | -1.12  (-2.68; 0.47) | -1.23  (-2.40; -0.04) | -2.14  (-3.29; -0.98) | -1.93  (-3.12; -0.73) | -1.49  (-2.66; -0.30) |
| **BC** | 0.31  (-0.63; 1.26) | 0.50  (-0.43; 1.44) | 0.49  (-0.44; 1.43) | 0.26  (-0.64; 1.17) | -0.39  (-0.65; -0.13) | -0.67  (-0.93; -0.42) | -0.63  (-0.90; -0.37) | -0.52  (-0.78; -0.26) |
| **Prevalence of prediabetes/diabetes** | | | | | | | | |
| **PM_2.5_** | 1.29  (1.04; 1.59) | 0.74  (0.58; 0.94) | 0.77  (0.60; 1.00) | 0.87  (0.67: 1.14) | 0.95  (0.78; 1.15) | 0.82  (0.67; 1.00) | 0.83  (0.67; 1.02) | 0.89  (0.72; 1.10) |
| **BC** | 1.07  (0.94; 1.23) | 1.13  (0.97; 1.31) | 1.13  (0.98; 1.31) | 1.11  (0.95; 1.23) | 0.97  (0.93; 1.01) | 0.93  (0.89; 0.97) | 0.93  (0.89; 0.98) | 0.95  (0.91; 0.99) |

*Results are expresses as percent change of 8-h fasting blood glucose concentration and odds ratio for prevalence of prediabetes/diabetes, and 95% confidence intervals (95%CI) per every 20 µg/m^3^ increase in personal fine particulate matter (PM_2.5_) and 1 µg/m^3^ increase in personal BC. Diabetes was defined as 8-h fasting blood glucose ≥7 mmol/l and/or participant either having self-reported diabetes or physician-diagnosed diabetes. Prediabetes was defined as 8-h fasting blood glucose ≥5.6 mmol/l and <7 mmol/l.*

1. Crude: outcome ~ predicted personal PM_2.5_/BC + (1 | village / household)
2. model P1: outcome ~ predicted personal PM_2.5_/BC + age + (1 | village / household)
3. model P2 (main model): model P1 + sugar and sweets intake + physical activity + education + alcohol intake
4. model P3: model P2 + body mass index + waist-to-hip-ratio + physician-diagnosed hypertension

**Table S5** Correlations and concentration means (in µg/m^3^) of the estimates of ambient and personal fine particulate matter (PM_2.5_) and black carbon (BC) according to relevant covariates.

|  | **AMBIENT** | | **PERSONAL** | |
| --- | --- | --- | --- | --- |
|  | PM_2.5_ | BC | PM_2.5_ | BC |
| **Continuous covariates (Spearman correlation)** | | | | |
| Age (years) | -0.02 | -0.02 | 0.29 | 0.10 |
| Physical activity | -0.09 | -0.02 | 0.30 | 0.24 |
| BMI | 0.06 | -0.03 | -0.09 | -0.01 |
| Waist-to-hip ratio | 0.05 | -0.01 | -0.11 | -0.21 |
| Alcohol intake | -0.11 | -0.01 | -0.09 | -0.22 |
| Sugar and sweets intake | 0.02 | -0.04 | -0.14 | -0.09 |
| **Categorical covariates (mean ± SD)** | | | | |
| Formal education |  |  |  |  |
| With any kind | 33.0 ± 2.6 | 2.5 ± 0.2 | 49.9 ± 10.1 | 5.1 ± 1.9 |
| Without | 32.8 ± 2.7 | 2.5 ± 0.2 | 59.3 ± 10.9 | 6.6 ± 2.7 |
| Smoking status |  |  |  |  |
| Current | 32.9 ± 2.5 | 2.5 ± 0.2 | 60.3 ± 7.2 | 4.4 ± 0.7 |
| Never or former | 32.9 ± 2.7 | 2.5 ± 0.2 | 53.5 ± 11.8 | 6.1 ± 2.6 |
| SLI |  |  |  |  |
| Low | 32.3 ± 2.7 | 2.5 ± 0.3 | 59.2 ± 11.5 | 6.9 ± 2.8 |
| Medium | 32.7 ± 2.5 | 2.5 ± 0.2 | 54.7 ± 11.3 | 5.8 ± 2.3 |
| High | 33.2 ± 2.6 | 2.5 ± 0.2 | 49.2 ± 9.3 | 4.5 ± 1.3 |
| ETS |  |  |  |  |
| No | 32.9 ± 2.7 | 2.5 ± 0.2 | 54.3 ± 11.1 | 5.6 ± 2.3 |
| Yes | 32.8 ± 2.5 | 2.5 ± 0.2 | 54.8 ± 12.3 | 6.3 ± 2.6 |
| Primary cooking fuel |  |  |  |  |
| Biomass | 32.5 ± 2.6 | 2.5 ± 0.2 | 58.4 ± 11.9 | 6.9 ± 2.6 |
| Clean | 33.0 ± 2.7 | 2.5 ± 0.2 | 48.7 ± 8.0 | 4.2 ±1.0 |

*BMI: body mass index; SD: Standard Deviation; ETS: Environmental Tobacco Smoke; SLI: Standard Living Index.*
